# The Effectiveness of Intravesical Oxybutynin, Propantheline, and Capsaicin in the Management of Neuropathic Bladder following Spinal Cord Injury

**DOI:** 10.1100/tsw.2007.271

**Published:** 2007-10-22

**Authors:** Jacob George, George Tharion, J. Richar, Ashish S. Macaden, Raji Thomas, Suranjan Bhattacharji

**Affiliations:** ^1^ Department of Physical Medicine and Rehabilitation, Christian Medical College, Vellore, Tamil Nadu, India; ^2^ Department of Biostatistics, Christian Medical College, Vellore, Tamil Nadu, India

**Keywords:** spinal cord injury, neurogenic detrusor overactivity, intravesical, oxybutynin, propantheline, capsaicin

## Abstract

The objective of this study was to compare the therapeutic response of intravesical oxybutynin, propantheline, and capsaicin in the treatment of neurogenic detrusor overactivity. Carried out in the Department of Physical Medicine and Rehabilitation at a university teaching hospital in India, patients acted as their own controls. Oxybutynin 5 mg in solution or propantheline 15 mg in solution and capsaicin were instilled intravesically in each patient. Urodynamic studies were done before and after the intravesical instillation of each drug. The nonparametric tests were used for statistical analysis. The efficacy of intravesical capsaicin in the treatment of neurogenic detrusor overactivity was statistically significant for reflex volume (RV) (p = 0.018), cystometric capacity (CC) (p = 0.0440), leak volume (LV) (p = 0.000), and leak frequency (LF) (p = 0.009). The Kruskal-Wallis test for paired sample comparing pre- and post-LV and LF for intravesical capsaicin was significant at 2^nd^ week (p = 0.002 and 0.054, respectively). There was a significant difference in therapeutic response between intravesical oxybutynin, propantheline, and capsaicin in the treatment of detrusor overactivity for LV and LF at 2^nd^ week (p = 0.017 and 0.003, respectively). When comparing responses of oxybutynin and propantheline, more subjects demonstrated improvement with intravesical propantheline than oxybutynin for RV, detrusor leak point pressure (LPP), clean intermittent catheterization volume (CICV), and LV. This study suggests that intravesical agents may be used as effective adjuvants in the management of incontinence due to neurogenic detrusor overactivity following spinal cord injury.

## INTRODUCTION

Neuropathic bladder following spinal cord injury (SCI) is a common clinical problem encountered in the management of patients with paraplegia and tetraplegia. Neurogenic detrusor overactivity (NDO) is a factor that contributes to urinary incontinence and reflux of urine, resulting in back pressure changes in the ureters and kidney. Clean intermittent catheterization (CIC) is the mainstay of management of neuropathic bladders aimed at low-pressure storage and timed emptying of the bladder. NDO can generate high pressures within the bladder, causing a deleterious effect on the bladder and kidneys. Systemic anticholinergics are widely prescribed to control overactive bladders to reduce intravesical pressure and improve continence. The systemic side effects of these agents can be unpleasant, causing patients to discontinue taking them. Locally active intravesical agents have been used with varying degrees of success in the management of overactive bladders[1]. Oxybutinin, an amino-acetyl ester derivative, is an anticholinergic with smooth muscle relaxant and local anesthetic properties. Propantheline bromide is a quartenary ammonium anticholinergic agent. Capsaicin (8 methyl-N-vannillyl-6-nonenamide) acts on vanilloid receptors of C-fiber sensory afferents, causing their desensitization by depleting the stores of neuropeptide, thereby suppressing sensory neuron activity[2,3,4]. In this article, we present our observations on the effectiveness of intravesical administration of oxybutynin, propantheline, and capsaicin in the management of neuropathic bladders following SCI.

## MATERIAL AND METHODS

Screening for NDO was done in 142 SCI patients with neuropathic bladders. Among them, 18 patients who fulfilled the selection criteria were included in the study. A written and informed consent was obtained from each patient after explaining the objectives of the study and the possible side effects. This study was approved by the institutional ethics committee.

### Inclusion Criteria

Patients with SCI of more than 3-months duration, age group 15–55 years, with normal upper urinary tract on ultrasound examination, who were on CIC and with overactive bladder on preliminary cystometric examination, were included in the study.

### Exclusion Criteria

Patients with intact urethral/bladder sensation or patients with recurrent symptomatic urinary tract infection or with past history of renal or urinary tract abnormality, or with psychiatric comorbidities were excluded.

### Evaluation of the Patient Prior to Starting Intravesical Drug Therapy

A detailed history was taken, followed by physical examination and neurological evaluation, including examination of perianal sensation and sacral reflexes according to the ASIA scale. Urine culture and sensitivity, upper urinary tract evaluation with ultrasound, cystoscopic evaluation of the lower urinary tract and urodynamic study for baseline detrusor pressure and compliance (Dantec Menuet Plus) were also performed. Bladder filling for cystometry was done at the rate of 20 ml/min[5].

### Trial Protocol

Oxybutynin 5 mg and propantheline 15 mg in solution was prepared by the hospital pharmacy and blinded. These where instilled intravesically three times daily for each patient by a double-blind method[6,7]. There was a 6-day washout period between each drug. As capsaicin is a long-acting drug that needs to be administered following local anesthesia, it was not possible to blind the capsaicin instillation. Urodynamic studies were done before and 3 h after the intravesical instillation of the third dose of either propantheline or oxybutynin. In the case of capsaicin, the urodynamic studies were repeated 1 and 2 weeks after instillation of the drug to allow for the initial local irritant effects of capsaicin to subside[8]. Data from the bladder diary maintained throughout the period of study was also analyzed.

## Instillation of Intravesical Oxybutynin and Propantheline

A commercially available, 15-mg tablet of propantheline bromide or 5 mg of oxybutynin hydrochloride was crushed and the powder dissolved in 10 ml of sterile water. These solutions were prepared by the pharmacy department of our institution, and both the investigator and the patient were blinded to the ingredient of the solution. The code of the blinded solutions was unmasked at the end of the study. This suspension was instilled into the bladder via a Foley's catheter after the patient had emptied the bladder by CIC. This solution remained in the bladder until it was emptied at the next CIC, which was done at regular intervals of 4–6 h. This procedure was carried out three times over a 24-h period at eight hourly intervals.

## Instillation of Intravesical Capsaicin

Capsaicin solution for intravesical use was made with a concentration of 1 m*M*/l in 30% ethanol in saline[9,10,11]. The bladder was emptied using a Foley's catheter. To prevent hyper-reflexic contractions, 40 ml of 2% lignocaine was instilled into the bladder and retained for 20 min prior to capsaicin administration[12]. The bladder was then emptied and a single dose of 100 ml of 1 m*M*/l capsaicin solution was instilled and retained for 30 min. Urodynamic studies were repeated 3 h after intravesical instillation followed by a repeat urodynamic study at 1 and 2 weeks.

## Parameters Observed after Intravesical Drug Administration

The subjective parameters compiled from the bladder diary were volume of urine passed at each CIC (CICV), number of times the CIC is performed, urine leak volume (LV) between CIC, and urine leak frequency (LF) between CIC.

The objective parameters obtained from urodynamics study were reflex volume**,** cystometric capacity, and detrusor leak point pressure. Reflex volume (RV) was defined as the volume at the start of the first detrusor overactivity contraction during filling cystometry. Cystometric capacity (CC) was defined as the volume infused during cystometry at which the patient can no longer delay micturition or, in the absence of sensation, the volume at which the bladder filling was terminated. Detrusor leak point pressure (LPP) was defined as the detrusor pressure required to induce leakage of urine through the urethra during cystometry. As patients in our study did not have any bladder sensation, we terminated the filling at 500 ml saline or earlier if leakage of saline through the urethra was observed.

Any adverse effects, like systemic anticholinergic effects, clinical urinary tract infection, and local bladder mucosal changes as observed by cystoscopy, were noted.

## Statistical Analysis

Nonparametric tests, Wilcoxon Signed Rank test, and Kruskal-Wallis test were used for statistical analysis. The statistical software used was SPSS (version 12).

## RESULTS

Among the 18 patients who participated in the study, there were 17 males and 1 female, age range: 20–53 years. The subjective as well as objective parameters were observed in all the participants.

Table 1 presents the mean, standard deviation (SD), and test of significance of comparison between pre- and postresponses of intravesical oxybutynin and propantheline for all parameters. This shows that there was significant worsening of LF following intravesical propantheline ($p$ = 0.039), whereas differences between pre- and postresponse for other parameters were not significant. The differences between pre- and postresponses for oxybutynin were also not significant for all parameters.

**TABLE 1**
**Subjective and Objective Changes in the Bladder following Instillation of Oxybutynin and Propantheline**

| Parameters | | Oxybutynin | | | Propantheline | | |
|---|---|---|---|---|---|---|---|
| | | Preinstil. | Postinstil. | *p** of Difference | Preinstil. | Postinstil. | *p** of Difference |
| RV | Mean | 154.3 | 154.8 | 0.744 | 131.9 | 120.2 | 0.760 |
| | SD | 96.5 | 106.8 | | 99.3 | 130.5 | |
| LPP | Mean | 61.3 | 64.4 | 0.443 | 68.7 | 76.7 | 0.532 |
| | SD | 51.3 | 53.1 | | 47.8 | 54.5 | |
| CC | Mean | 257.2 | 237.4 | 0.407 | 206.3 | 190.9 | 0.495 |
| | SD | 151.4 | 179.0 | | 169.4 | 195.1 | |
| CICV | Mean | 186.4 | 176.9 | 0.121 | 176.9 | 180.6 | 0.499 |
| | SD | 69.7 | 57.4 | | 57.4 | 66.3 | |
| LV | Mean | 92.8 | 111.3 | 0.279 | 111.3 | 131.3 | 0.382 |
| | SD | 133.0 | 108.3 | | 108.3 | 134.8 | |
| LF | Mean | 3.4 | 4.5 | 0.126 | 4.5 | 5.6 | 0.039 |
| | SD | 3.6 | 4.1 | | 4.1 | 5.0 | |

*Wilcoxon – Signed rank test.

RV, reflex volume in ml; LPP, detrusor leak point pressure in cm water; CC, cystometric capacity in ml; CICV, clean intermittent catheterization volume in ml; LV, leak volume between clean intermittent catheterization in ml; LF, urine leak frequency (number of leaks per day).

Table 2 gives the mean, SD, and test of significance of comparison between pre- and postresponses of intravesical capsaicin for all parameters. This shows that the overall differences (Column A) were significant in response to RV, CC, LV, and LF ($p$ = 0.018, 0.044, 0.000, and 0.009, respectively). When comparing the differences between pre- and 3 h postcapsaicin (Column B), objective parameters like RV and CC significantly worsened ($p$ = 0.008 and 0.015, respectively). However, the differences between pre- and 2 weeks postcapsaicin with respect to subjective parameters like LV and LF showed significant improvement. ($p$ = 0.002 and 0.054, respectively).

Table 3 shows the results of the Kruskal-Wallis test comparing differences in pre- and postresponses between intravesical oxybutynin, propantheline, and capsaicin. This shows that there were significant differences in pre- and postresponses between oxybutynin, propantheline, and capsaicin at 2 weeks with respect to LV and LF ($p$ = 0.017 and 0.003, respectively).

**TABLE 2**
**Mean, SD, and Test of Significance* of Changes in the Bladder following Instillation with Capsaicin**

| Parameters | | Pre | 3 h | 1 week | 2 weeks | *p* of Difference | | | |
|---|---|---|---|---|---|---|---|---|---|
| | | | | | | A | B | C | D |
| RV | Mean | 54.3 | 85.6 | 125.8 | 183.4 | 0.018 | 0.008 | 0.198 | 0.199 |
| | S.D. | 120.8 | 61.4 | 80.0 | 130.9 | | | | |
| LPP | Mean | 66.8 | 77.4 | 77.4 | 62.1 | 0.506 | 0.605 | 0.163 | 1.000 |
| | S.D. | 43.4 | 46.6 | 41.6 | 41.3 | | | | |
| CC | Mean | 224.6 | 139.6 | 174.2 | 249.9 | 0.044 | 0.015 | 0.059 | 0.495 |
| | S.D. | 171.4 | 146.0 | 145.2 | 159.6 | | | | |
| CICV | Mean | 180.6 | 163.9 | 163.9 | 180.6 | 0.730 | 0.106 | 0.106 | 0.476 |
| | S.D. | 66.3 | 65.1 | 65.1 | 66.3 | | | | |
| LV | Mean | 131.3 | 180.4 | 180.4 | 38.4 | 0.000 | 0.096 | 0.096 | 0.002 |
| | S.D. | 134.8 | 148.8 | 148.8 | 59.8 | | | | |
| LF | Mean | 5.6 | 7.1 | 7.1 | 3.3 | 0.009 | 0.80 | 0.080 | 0.054 |
| | S.D. | 5.0 | 4.1 | 4.1 | 5.3 | | | | |

*Kruskal – Wallis test (using individual differences).

A, overall difference; B, between pre and 3 h post; C, between pre and 1 week post; D, between pre and 2 weeks post.

**TABLE 3**
**Results of Kruskal-Wallis Test Comparing Difference in Pre- and Postinstillation Responses between Oxybutynin, Propantheline, and Capsaicin**

| Therapeutic Response Compared | | 3 h | 1 week | 2 weeks |
|---|---|---|---|---|
| RV | $\chi^2$ | 3.580 | 0.213 | 1.954 |
| | *p* | 0.167 | 0.899 | 0.377 |
| LPP | $\chi^2$ | 0.003 | 0.952 | 0.104 |
| | *p* | 0.998 | 0.621 | 0.949 |
| CC | $\chi^2$ | 2.155 | 0.675 | 1.616 |
| | *p* | 0.340 | 0.714 | 0.446 |
| CICV | $\chi^2$ | 4.656 | 4.656 | 2.390 |
| | *p* | 0.097 | 0.097 | 0.303 |
| LV | $\chi^2$ | 0.548 | 0.548 | 8.188 |
| | *p* | 0.760 | 0.760 | 0.017 |
| LF | $\chi^2$ | 1.262 | 1.262 | 11.430 |
| | *p* | 0.532 | 0.532 | 0.003 |

Table 4 presents the number of patients who demonstrated improvement in response to intravesical oxybutynin, propantheline, and capsaicin at 3 h, 1 week, and 2 weeks. It was observed that more subjects demonstrated improvement with propantheline than oxybutynin for RV, LPP, CICV, and LV. There was marked progressive overall improvement for parameters, except LPP, following capsaicin.

**TABLE 4**
**Number of Subjects who Showed Improvement in Response to Oxybutynin, Propantheline, and Capsaicin**

| Response | Oxybutynin | Propantheline | Capsaicin 3 h | Capsaicin 1 week | Capsaicin 2 weeks |
|---|---|---|---|---|---|
| | N (%) | | | | |
| RV | 7 (39) | 8 (44) | 4 (22) | 7 (39) | 11 (61) |
| LPP | 6 (33) | 7 (39) | 8 (44) | 5 (28) | 6 (33) |
| CC | 6 (33) | 6 (33) | 4 (22) | 6 (33) | 9 (50) |
| CICV | 4 (22) | 10 (56) | 3 (17) | 3 (17) | 6 (33) |
| LV | 4 (22) | 5 (28) | 2 (11) | 3 (17) | 12 (65) |
| LF | 4 (22) | 4 (22) | 3 (17) | 3 (17) | 10 (56) |

During the study, two patients complained of increased thirst and dryness of mouth following intravesical oxybutynin, and two patients had symptoms of autonomic dysreflexia following instillation of capsaicin. This was dealt with with sublingual nifedipine. None of them had clinical urinary tract infection.

## DISCUSSION

Strategies for treating NDO by administration of intravesical agents are based on the principles of either inhibiting parasympathetic efferent activity or blocking the sensory afferents from the bladder[13]. NDO may cause urinary leakage, upper urinary tract deterioration, or vesicoureteral reflux. The goal of treatment is to achieve continence with a low-pressure system and to ensure adequate bladder emptying[14].

Adequate bladder emptying can be achieved by CIC, but urinary incontinence in between may persist in the presence of detrusor overactivity. Pharmacological relaxation of the detrusor together with CIC is an accepted method to prevent urinary incontinence. Systemic anticholinergics that relax the bladder cause adverse effects like dry mouth and blurred vision. Instillation of the drug into the bladder reduces the systemic side effects and the intravesical application is a logical approach, especially for patients already on CIC, as the drug can conveniently be instilled regularly at the end of catheterization without any additional instrumentation[13].

Common intravesical medications that block detrusor cholinergic activity are propantheline and oxybutynin. Use of the botulinum-A toxin, which presumably acts at the neuromuscular junction (mechanism of action is not well known), is also a common method. Inhibiting transmission from the bladder along afferent C-fibers by intravesical administration of compounds collectively described as vanilloids is another attractive option. Such agents that block the afferent arm of the reflex arc include capsaicin and resiniferatoxin. Capsaicin exerts its durable beneficial effects in about 60–70% of patients. Intravesical administration of these agents can reduce the systemic side effects and concurrently optimize the beneficial effects[15,16]. There are several studies on the effectiveness of intravesical oxybutynin and capsaicin, but there is a paucity of literature on the effects of intravesical propantheline. Beneficial effects of intravesical propantheline in experimental animals have been reported[17,18,19].

In a study among 13 patients with complete suprasacral spinal cord lesions, Madersbacher reported a statistically significant increase in CC and reduction in maximum detrusor pressure following intravesical oxybutynin[13]. In a study among 15 patients, O'Flynn and Thomas observed that 12 patients had a reduction in the frequency and amplitude of overactive contractions and incontinence episodes following intravesical oxybutynin administration[20]. Szollar and Lee observed that, among 13 SCI patients who were treated with intravesical oxybutynin, nine patients showed improvement in bladder capacity and LPP[21]. However, in our study, there were no statistically significant changes in these parameters following intravesical oxybutynin.

Treatment with intravesical propantheline did not produce any statistically significant change in any of the parameters except for a significant worsening of the LF. It was interesting to note that following intravesical propantheline instillation, there was a fall in detrusor LPP in seven patients, and an increase in RV in eight patients and CC in six patients. Subjective parameters like CIC volume increased in 10 patients, LF and LV decreased in four and five patients, respectively. Even though the bladder volume at CIC increased in 10 patients, the differences were not statistically significant (Table 1).

Three hours following intravesical administration of capsaicin, cystometry as well as the bladder diary demonstrated that there was deterioration of all the parameters. This could be due to the early effects of capsaicin causing bladder irritation, which are ascribed to initial stimulation before C-fiber inactivation. However, when these parameters were observed 2 weeks after capsaicin instillation, there was a statistically significant decrease in LV and LF, ($p$ = 0.002 and 0.054, respectively; Table 2, column D). Although there was an improvement in CC among 50% of patients, it was not statistically significant. It is possible that administration of capsaicin at a higher concentration would have shown improvement in these parameters.

Lazzeri et al. observed the urodynamic effects of intravesical capsaicin infusion with real-time cystometrogram in 30 patients with detrusor overactivity following chronic spinal myelopathy. There was clinical and urodynamic improvement in CC among 15 of the 30 patients (50%). The improvement of CC in our study was also similar (50%), but this was not a real-time infusion study[22]. In a study among 13 patients with intravesical capsaicin for detrusor overactivity, Dasgupta et al. observed improvement in CC in nine patients[23]. Fowler et al. also reported improvement in CC in nine patients following intravesical instillation of capsaicin among 14 patients[24]. In our study, there was an overall statistically significant response to RV, CC, LV, and LF ($p$ = 0.018, 0.044, 0.000, and 0.009, respectively; Table 2, column A) following intravesical capsaicin. It has been suggested that intravesical treatment strategies in patients with NDO may provide alternatives to established therapies, such as oral anticholinergics. The selectivity of intravesical treatments and the reduction of systemic side effects are the major advantages of these topical agents.

## CONCLUSION

There was statistically significant improvement in LV and LF at 2 weeks following intravesical capsaicin. It was also observed that more subjects demonstrated improvement with intravesical propantheline than oxybutynin for RV, detrusor LPP, CICV, and LV. This study suggests that intravesical agents may be used as effective adjuvants in the management of incontinence due to overactive neuropathic bladders following SCI.

## ACKNOWLEDGMENTS

We thank the CMC Fluid Research Grants Committee for funding this study. We also thank Dr. Anna Durai (Department of Pharmacy Services, CMC) for help in conducting this study and manuscript preparation.

This study was funded by the Indian Council of Medical Research (IRIS ID No.9701730).
